# Molecular Mimicry Between Epstein‐Barr Virus and Human Herpesvirus‐6 Proteins and Central Nervous System Proteins: Implications for T and B Cell Immunogenicity in an In Silico Study

**DOI:** 10.1002/iid3.70445

**Published:** 2026-05-13

**Authors:** Abbas F. Almulla, Muslimbek G. Normatov, Thitiporn Supasitthumrong, Michael Maes

**Affiliations:** ^1^ Sichuan Provincial Center for Mental Health, Sichuan Provincial People's Hospital, School of Medicine University of Electronic Science and Technology of China Chengdu China; ^2^ Key Laboratory of Psychosomatic Medicine Chinese Academy of Medical Sciences Chengdu China; ^3^ Medical Laboratory Technology Department, College of Medical Technology The Islamic University Najaf Iraq; ^4^ Department of Pathology, Faculty of Medicine Saint Petersburg State University Saint Petersburg Russia; ^5^ Department of Psychiatry, Faculty of Medicine Chulalongkorn University Bangkok Thailand; ^6^ Cognitive Fitness and Technology Research Unit, Faculty of Medicine Chulalongkorn University Bangkok Thailand; ^7^ Department of Psychiatry Medical University of Plovdiv Plovdiv Bulgaria; ^8^ Research Institute Medical University Plovdiv Plovdiv Bulgaria; ^9^ Kyung Hee University Seoul Republic of Korea

**Keywords:** autoimmunity, CNS, EBV, HHV‐6, molecular mimicry, neuropsychiatric disorders

## Abstract

**Background:**

The Epstein‐Barr virus (EBV) and human herpesvirus 6 (HHV‐6) are frequently linked to neuropsychiatric illnesses such as multiple sclerosis, depression, and chronic fatigue syndrome/myalgic encephalomyelitis. These viruses may induce autoimmune reactions by molecular mimicry, leading to damage to self‐epitopes in the central nervous system (CNS).

**Objective:**

This study seeks to explore the common pentapeptides present in EBV and HHV‐6 viral antigens alongside various CNS‐related proteins via molecular mimicry. Additionally, it will assess the immunogenicity of these shared pentapeptides in T and B cells.

**Method:**

Sequence alignment was conducted to assess molecular mimicry between 32 EBV and HHV‐6 antigens and 10 CNS autoantigens. Protein sequences were obtained from UniProt, structural homology was analyzed using AlphaFold and PyMol, and shared pentapeptides were identified with Alignmentaj. Immunogenicity was assessed via the Immune Epitope Database (IEDB) for potential T‐ and B‐cell activation.

**Results:**

A total of 91 mimicry pentapeptides were identified between viral antigens (42 EBV and 49 human HHV‐6), and 10 CNS proteins. Notably, synapsin (SYN)1 exhibited the highest mimicry, sharing 13 pentapeptides with (7 with EBV and 6 with HHV‐6) viral antigens such as EBV nuclear antigen (EBNA)1, EBNA6, latent membrane protein (LMP)1, and early antigen diffused (EA‐D). Myelin proteins, including myelin‐associated glycoprotein with 12 shared pentapeptides, myelin basic protein with 9, and myelin‐oligodendrocyte glycoprotein with 5, displayed immune cross‐reactivity with EBV/HHV‐6 antigens. EBNA1, EBNA2, EBNA6, LMP1, LMP2, EA‐D, and BLLF1 structurally resemble CNS autoantigens and act as immunoreactive epitopes for human T and B cells. Except for EBNA2 and protein U94, all share immunogenic pentapeptide sequences with SYN1.

**Conclusion:**

Shared pentapeptides suggest a link between viral infections and CNS autoimmunity. Further research is needed to clarify molecular mechanisms and explore targeted therapies to mitigate virus‐induced neuroinflammation.

## 1 Introduction

Molecular mimicry, the phenomenon where foreign antigens from pathogens exhibit structural resemblance to self‐antigens, is a significant contributor to autoimmune responses [1, 2]. Structurally similar antigens, when presenting to the immune system, are able to generate immune responses mistakenly targeting host tissues, leading to damage as a result of the inability of the immune system to distinguish self from non‐self‐antigens [2, 3, 4]. In central nervous system (CNS) diseases such as multiple sclerosis (MS), where the autoreactive T cells are known to target myelin proteins, molecular mimicry was frequently reported as a key mechanism [5].

Viruses and bacteria contain antigens that resemble myelin proteins, leading to cross‐reactive immune responses [4, 6]. A growing body of evidence linked infections caused by viruses like Epstein‐Barr virus (EBV), human herpesvirus 6 (HHV‐6), Severe acute respiratory syndrome coronavirus 2 (SARS‑CoV‑2), and Zika virus (ZikV) with autoimmune CNS diseases, including but not limited to MS [7, 8, 9]. França et al. reported an association between Zika virus (ZikV) infection and neurological complications, with its NS5 protein mimicking CNS antigens and triggering autoimmune responses [7]. Additionally, SARS‐CoV‐2 has been linked to autoimmune diseases in genetically susceptible individuals, further underscoring the impact of viral infections on CNS pathology [8]. These findings underscore the viruses' potential to trigger autoimmune demyelinating processes via molecular mimicry.

Recent findings suggest that EBV and HHV‐6 reactivation may play a significant role in sustaining autoimmune processes in MS patients, where autoantibodies targeting myelin proteins are linked to viral reactivation [10]. These results demonstrated that autoantibodies against myelin proteins, such as myelin basic protein (MBP) and myelin oligodendrocyte glycoprotein (MOG), in those patients are associated with reactivation of EBV and HHV‐6 [10]. Similarly, in Long COVID disease, our results revealed significant correlations between HHV‐6 reactivation and autoantibodies (immunoglobulins A and G) targeting zonulin and occludin, which are tight junction proteins. HHV‐6 and EBV reactivation and consequent autoimmune responses to CNS self‐epitopes may play a role in Long COVID, depression, chronic fatigue syndrome/myalgic encephalomyelitis (CFS/ME) [11, 12, 13, 14]. The findings emphasize that these viruses' reactivation has key roles in triggering or sustaining autoimmune responses [12].

CNS proteins, including MBP, MOG, and proteolipid (PLP), are essential for maintaining myelin integrity and neural conduction [15, 16]. Disruption of these proteins through immune‐mediated attacks results in demyelination and impaired neuronal function, as noted in MS [17]. Tight junction proteins such as claudin‐5 and occludin, which maintain blood‐brain barrier (BBB) integrity alongside synaptic protein such as synapsins, are also targeted in autoimmune CNS disorders, exacerbating inflammation and neurodegeneration [18, 19, 20]. Molecular mimicry is a key mechanism in CNS diseases, though other factors, such as epitope spreading and immune‐inflammatory responses, also contribute [9, 21, 22]. Miller et al. reported that T cell responses targeting the immunodominant myelin PLP‐139‐151 epitope did not result from cross‐reactivity between Theiler's murine encephalomyelitis virus and self‐epitopes, commonly referred to as molecular mimicry. Instead, these responses were attributed to de novo priming of self‐reactive T cells to sequestered autoantigens released as a consequence of virus‐specific T cell‐mediated demyelination, known as epitope spreading [23].

EBV and HHV‐6 exhibit neurotrophic properties that substantially influence CNS disease. These viruses establish latency in host cells, avoid immune surveillance, and periodically reactivate, presenting continuous threats to brain health [24, 25]. Lanz et al. revealed a molecular mimicry between EBV antigen (namely EBV transcription factor EBV nuclear antigen 1, EBNA1 and glial cell adhesion molecule (GlialCAM) in MS disease [26]. HHV‐6 can impair cellular immunity by targeting CD4 + T cells, further complicating immune dysregulation [27]. Likewise, Mameli et al. reported that HHV‐6 antigens mimic MBP resulting in autoimmune responses against the latter [28]. Moreover, EBV and HHV‐6 viral proteins mimic important CNS proteins like α and β‐ synuclein, neurofilament light chain [29, 30], anoctamin 2 [31], α‐B crystallin (CRYAb) [32] and Septin‐9 [33], suggesting contributions of these mechanisms in the pathogenesis of neuroautoimmune diseases. Kanduc (2013) reported that pentapeptides can induce highly specific antibodies and mediate precise immune interactions [34]. However, our understanding of mimicry across a broader spectrum of CNS proteins is still not fully comprehended, as most research focuses on a limited range of proteins.

This study investigates the molecular mimicry of similar pentapeptides between 32 EBV and HHV‐6 proteins and various CNS proteins, including myelin proteins (MBP, MOG, PLP), BBB proteins (occludin, claudin‐5), cytoskeletal proteins (tubulin), and synaptic proteins (synapsin). We will assess the immunogenicity of these shared pentapeptides to T‐ and B‐cells. The central hypothesis suggests that particular amino acid sequences in viral proteins structurally mimic CNS proteins, eliciting immune responses that may contribute to autoimmune and neuroinflammatory disorders, including MS.

## 2 Materials and Methods

### 2.1 Data Retrieval

To examine molecular mimicry between herpesviruses (EBV and HHV‐6) antigens and CNS autoantigens, 32 immunogenic viral antigens were selected. The EBV antigens include EBNA1, EBNA2, EBNA6 Latent Membrane Protein (LMP) 1, LMP2A/LMP2B, Early Antigen‐Diffuse (EA‐D), Early Antigen‐Restricted (EA‐R), Epstein‐Barr Virus Glycoprotein gp350/220 (BLLF1), Glycoprotein B (gB), Glycoprotein H (gH), Major Capsid Protein (MCP), and Glycoprotein E (gE). The HHV‐6 antigens include immediate‐early protein 2 (U90/U87/U86), MCP, ribonucleotide reductase large subunit (RIR1), U24 protein, 120 kDa Glycoprotein O (U47), Large structural phosphoprotein (U11), DNA binding protein (DBP), putative CC‐type chemokine (U83), DNA polymerase catalytic subunit (U38), capsid scaffolding protein (U53), large tegument protein deneddylase (U31), gH, glycoprotein (U21), Glycoprotein Q2 (U100), gB, G‐protein coupled receptor homolog (U12), Glycoprotein 105 (U96/U97/U98/U99/U100), Uracil‐DNA glycosylase (U81), G‐protein coupled receptor homolog (U51), and U94 protein.

These viral proteins were examined for their structural homologies (shared pentapeptides) with 10 selected human CNS autoantigens (MBP, MOG, MOBP, MAG, SYN1, SYN2, CLDN5, OCLN, TUBA1A and TUBB), which serve as potential targets in the autoimmune neuropathies observed in MS, ME/CFS, and affective syndromes. The selected autoantigens were chosen to represent major structural and functional components of the CNS that are implicated in neuroautoimmune and neuroinflammatory disorders. These include myelin‐associated proteins (MBP, MOG, MOBP, MAG), synaptic proteins (SYN1, SYN2), and proteins involved in blood–brain barrier integrity and neuronal cytoskeletal structure (CLDN5, OCLN, TUBA1A, TUBB). Pentapeptide matches were defined as identical five‐amino‐acid motifs shared between viral proteins and human CNS proteins.

The amino acid sequences of EBV and HHV‐6 antigens and human CNS autoantigens were obtained from the Uniprot database [35] in fasta format. The ID number of EBV and HHV‐6 antigens along with human CNS autoantigens in the UniProt database is shown in Table 1.

**Table 1:** UniProt code for Epstein–Barr virus (EBV), Human herpesvirus 6 (HHV‐6) and central nervous system (CNS) antigens utilized in the current study.

	No.	Viral antigen	Code in UniProt	CNS protein	Code in UniProt
Epstein–Barr virus	1	EBNA1	P03211	MBP	P02686
	2	EBNA2	P12978	MOG	Q16653
	3	LMP1	P03230	MAG	P20916
	4	LMP2A/LMP2B	P13285	SYN1	P17600
	5	EA‐D (BMRF1)	P03191	SYN2	Q92777
	6	BLLF1	P03200	OCLN	Q16625
	7	gB	P03188	CLDN5	O00501
	8	gH	P03231	TUBB	P07437
	9	MCP	P03226	TUBA1A	Q71U36
	10	EBNA6	P03204	MOBP	Q13875
	11	gE	P09259		
	12	BHRF1	P03182		
Human herpesvirus 6	13	Glycoprotein Q2 (U100)	P0DOE0		
	14	Capsid scaffolding protein (U53)	P24433		
	15	gB	P36319		
	16	U24 protein	Q69559		
	17	Immediate‐early protein 2 (U90/U87/U86)	Q77Z83		
	18	Large tegument protein deneddylase (U31)	P30002		
	19	G‐protein coupled receptor homolog (U12)	P52380		
	20	gH	P68324		
	21	120 kDa Glycoprotein O (U47)	Q06093		
	22	Glycoprotein 105 (U96/U97/U98/U99/U100)	Q69489		
	23	Glycoprotein U21 (U21)	Q69556		
	24	Immediate‐early protein 2 (U90/U86)	Q8BB47		
	25	MCP	P17887		
	26	DNA polymerase catalytic subunit (U38)	P28857		
	27	DBP	P52338		
	28	RIR1	P52343		
	29	Uracil‐DNA glycosylase (U81)	P52345		
	30	G‐protein coupled receptor homolog (U51)	P52382		
	31	Putative CC‐type chemokine (U83)	P52460		
	32	U94 Protein	Q00683		
	33	Large structural phosphoprotein (U11)	Q00701		

Abbreviations: BLLF1, envelope glycoprotein GP350; CLDN5, claudin‐5; DBP, major DNA‐binding protein; EA‐D, early antigen diffused; EA‐R, early antigen protein R; EBNA, Epstein–Barr nuclear antigen; gB, envelope glycoprotein B; gE, envelope glycoprotein E; gH, envelope glycoprotein H; LMP, latent membrane protein; MBP, myelin basic protein; MCP, major capsid protein; MOBP, myelin‐associated oligodendrocyte basic protein; MOG, myelin‐oligodendrocyte glycoprotein; OCLN, occludin; RIR1, ribonucleoside‐diphosphate reductase large subunit‐like; SYN, synapsin; TUBA1A, tubulin alpha‐1A chain; TUBB, tubulin beta chain.

The AlphaFold database [36] and the PyMol program [37] (https://www.pymol.org/) were used to illustrate the location of mimicry pentapeptides in the 3D surface structures of human nervous system autoantigens. All 3D structures of the human nervous system auto‐antigens were obtained in PDB format from the AlphaFold database. The ID number of 3D structure of human nervous system autoantigens in the AlphaFold database is indicated in Table 1.

### 2.2 Sequence Alignment (Definition of Mimicry Pentapeptides)

Our original program ‘Alignmentaj’ (https://github.com/muslimb/MyProekt1; Muslimbek), registered by the Russian Federal Agency for Intellectual Property (Certificate No. 2023617186, April 6, 2023), was used to identify identical pentapeptide motifs shared between viral (EBV and HHV‐6) and human proteins (MBP, MOG, MOBP, MAG, SYN1, SYN2, CLDN5, OCLN, TUBA1A and TUBB). Unlike conventional alignment tools such as BLASTp, which primarily detect extended regions of sequence homology, Alignmentaj performs systematic scanning of overlapping five‐amino‐acid windows, allowing reliable detection of short identical motifs relevant for molecular mimicry analyzes. As input, the program receives the amino acid sequences of human antigens and viral antigens in fasta format. The program divides the amino acid sequences of the virial antigens into pentapeptides (i.e., MSDEG, SDEGP, DEGPG, and so forth) and the program aligns the pentapeptides to the amino acid sequences of the human autoantigen. Pentapeptides, utilized, as five‐residue sequences constitute minimal functional and immunologically relevant units, thereby offering a biologically valid foundation for detecting potential molecular mimicry between pathogen and host proteins [38]. Experimental studies have demonstrated that a pentapeptide motif can represent the minimal antigenic determinant presented by MHC class I molecules and recognized by cytotoxic T cells, highlighting the potential of very short peptide motifs to mediate specific immune recognition [39]. Hemmer et al. found that autoreactive CD4⁺ T‐cell clones have shown that pentapeptides and even shorter peptides can stimulate T‐cell responses and improve T‐cell survival. This demonstrates the great potential for cross‐reactivity between short peptide motifs and the degeneracy of T‐cell receptor recognition [40]. The output of the program shows existing mimicry pentapeptides between human autoantigens and microbial antigens.

### 2.3 Analysis of Pentapeptide Mimicry Between EBV, HHV‐6, and CNS Antigens for T and B Cell Immunogenicity

To investigate the immunogenic potential of the shared pentapeptides found in viral antigens (EBV and HHV‐6) and different CNS proteins in stimulating T and B cells, we performed molecular mimicry analyzes of these shared pentapeptides in relation to T and B cell activation. The Immune Epitope Database (IEDB) [41] was utilized to investigate the immunogenicity of mimicking pentapeptides of EBV and HHV‐6 in relation to CNS antigens, focusing specifically on human immune cells, including T and B cells. The IEDB compiles experimental data regarding antibody and T cell epitopes that have been investigated in human subjects.

## 3 Results

### 3.1 The Results of Molecular Mimicry Between EBV and HHV‐6 Viral Antigens and CNS Proteins

#### 3.1.1 EBV Mimicry

**Figure 1:**
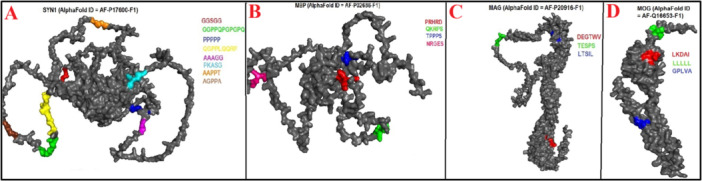
(A–D). Localization of Epstein–Barr virus (EBV)–mimicking pentapeptides mapped onto AlphaFold‐predicted 3D structures of synapsin I (SYN1), myelin basic protein (MBP), myelin‐associated glycoprotein (MAG), and myelin oligodendrocyte glycoprotein (MOG).

In the present study, a total of 42 mimicry pentapeptides were identified between EBV and HHV‐6 virial antigens and CNS antigens. As shown in Table 2, four mimicry pentapeptides were identified between EBNA1 and SYN1, while the same CNS proteins (SYN1) showed 3 mimicry pentapeptide with and EBNA6 (see Figure 1A). This CNS protein also showed 2 mimicry pentapeptide with LMP1 and EA‐D viral antigens (see Figure 1A). Furthermore, only 1 mimicry pentapeptide was observed between SYN1 and EBNA2 and LMP2A/LMP2B as shown in Figure 1A. Myelin proteins, namely MBP (Figure 1B) and MAG (Figure 1C), showed two mimicry pentapeptides with EBNA1, while MAG showed one mimicry pentapeptide with EBNA2 and gE (see Figure 1C). MBP showed one single mimicry pentapeptide with EBNA6 and gE as presented in Figure 1B. Similarly, as displayed in Figure 1D, MOG showed one single mimicry pentapeptide with EBNA1, LMP1, BLLF1, and EBNA6.

**Table 2:** Results of molecular mimicry between Epstein–Barr virus (EBV), Human herpesvirus 6 (HHV‐6) and central nervous system (CNS) antigens.

Virus	EBV and HHV‐6 antigens	Human CNS autoantigens	Similar pentapeptides between Epstein‐Barr virus, Human herpesvirus 6 antigens and human CNS autoantigens	^#^Total shared pentapeptide
EBV	EBNA1	MBP	PRHRD, QKRPS	2
		MOG	LKDAI	1
		MAG	DEGTW, EGTWV	2
		SYN1	GGSGG, PGPGP, GPGPQ, PQPGP	4
		SYN2	RRPPP	1
	EBNA2	MAG	TESPS	1
		SYN1	PPPPP	1
		SYN2	PPPPP, DYIFE	2
		OCLN	PPGVI	1
	LMP1	MOG	LLLLL	1
		SYN1	GGPPQ, QGPPL	2
	LMP2A/LMP2B	SYN1	AAAGG	1
		CLDN5	LLAFV, LGAAL	2
	EA‐D	SYN1	LQQRP, PPPPP	2
		SYN2	PPPPP	1
	BLLF1	MOG	LLLLL	1
		MOBP	AVVRA	1
		SYN1	PKASG	1
	gB	TUBB	LTTPT	1
	gH	TUBA1A	SLRFD	1
	MCP	OCLN	KRTEQ, SPMAY	2
	EBNA6	MBP	TPPPS	1
		MOG	GPLVA	1
		SYN1	PGPGP, AAPPT, AGPPA	3
		SYN2	AAPPT, SSSAP	2
	gE	MBP	NRGES	1
		SYN2	YAGLP	1
		OCLN	PVDDF	1
		MAG	LTSIL	1
	BHRF1	—	—	—
HHV‐6	Immediate‐early protein 2 (U90/U87/U86)	SYN2	SSSSA, GEKFP, EKFPL	3
		OCLN	VKNVS, SKRAP	2
		MAG	RISGA	1
	MCP	MAG	LTSIL, EVEVS	2
		MBP	LSKIF	1
		SYN2	AARKA	1
		OCLN	PITSD	1
	RIR1	OCLN	DKELD, KELDD	2
		MAG	TVGLS	1
		MBP	ASQKR	1
	U24 protein	MBP	PRTPP, RTPPP, TPPPS	3
	120 kDa Glycoprotein O (U47)	TUBA1A	SILTT, ATIKT	2
		SYN1	LSSTT	1
	Large structural phosphoprotein (U11)	MAG	SNVSP, VAESL	2
		SYN2	TLGGE	1
	DBP	TUBB	LVDSV, LNHLV	2
	Putative CC‐type chemokine (U83)	CLDN5	GCLLC, CLLCC	2
	DNA polymerase catalytic subunit (U38)	MBP	FKGVD	1
		SYN1	EVKAE	1
		CLDN5	GVLYL	1
	Capsid scaffolding protein (U53)	MBP	GLSLS	1
		MAG	RDTVQ	1
	Large tegument protein deneddylase (U31)	MAG	AFELP	1
		SYN2	KQTAA	1
	gH	MBP	YLATA	1
		MAG	ILIAI	1
		SYN1	YAGIP	1
	Glycoprotein (U21)	SYN1	GSAML	1
		SYN2	GSAML	1
	Glycoprotein Q2 (U100)	MAG	ALPLF	1
	gB	TUBB	TDETY	1
	G‐protein coupled receptor homolog (U12)	MAG	LCSTQ	1
	Glycoprotein 105 (U96/U97/U98/U99/U100)	MOG	LIFLC	1
		SYN1	AFSMA	1
	Uracil‐DNA glycosylase (U81)	TUBB	PSPKV	1
	G‐protein coupled receptor homolog (U51)	SYN2	NNYKA	1
	U94 Protein	SYN1	HSPGA	1
		OCLN	SLLGG	1
	**Total**	**91 (42 EBV, 49 HHV‐6)**		

Abbreviations: BLLF1, envelope glycoprotein GP350; CLDN5, claudin‐5; DBP, major DNA‐binding protein; EA‐D, early antigen diffused; EA‐R, early antigen protein R; EBNA, Epstein–Barr nuclear antigen; gB, envelope glycoprotein B; gE, envelope glycoprotein E; gH, envelope glycoprotein H; LMP, latent membrane protein; MBP, myelin basic protein; MCP, major capsid protein; MOBP, myelin‐associated oligodendrocyte basic protein; MOG, myelin‐oligodendrocyte glycoprotein; OCLN, occludin; RIR1, ribonucleoside‐diphosphate reductase large subunit‐like; SYN, synapsin; TUBA1A, tubulin alpha‐1A chain; TUBB, tubulin beta chain.

**Figure 2:**
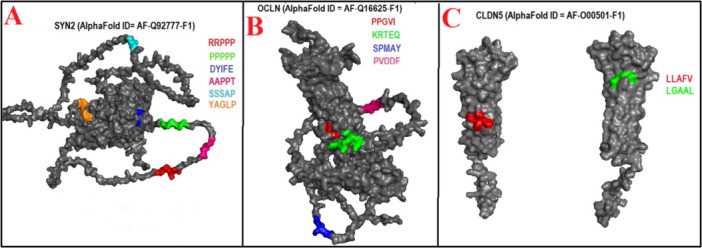
(A–C). Localization of Epstein–Barr virus (EBV)–mimicking pentapeptides mapped onto AlphaFold‐predicted 3D structures of synapsin II (SYN2), occludin (OCLN), and claudin‐5 (CLDN5).

**Figure 3:**
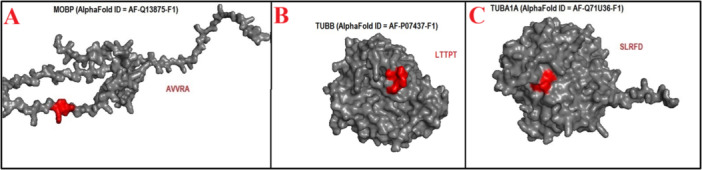
(A–C). Localization of Epstein–Barr virus (EBV)–mimicking pentapeptides mapped onto AlphaFold‐predicted 3D structures of myelin‐associated oligodendrocyte basic protein (MOBP), tubulin beta chain (TUBB), and tubulin alpha‐1A chain (TUBA1A).

Figure 2A showed that SYN2 shared two mimicry pentapeptides with EBNA2 and EBNA6, and one single mimicry pentapeptide with EBNA1, EA‐D, and gE. OCLN (Figure 2B) showed two mimicry pentapeptides with MCP and single one with EBNA2 and gE. CLDN5 (Figure 2C), MOBP (Figure 3A), TUBB (Figure 3B), and TUBA1A (Figure 3C) shared single mimicry pentapeptides with EBNA2, BLLF1, gB, and gH respectively.

#### 3.1.2 HHV‐6 Mimicry

**Figure 4:**
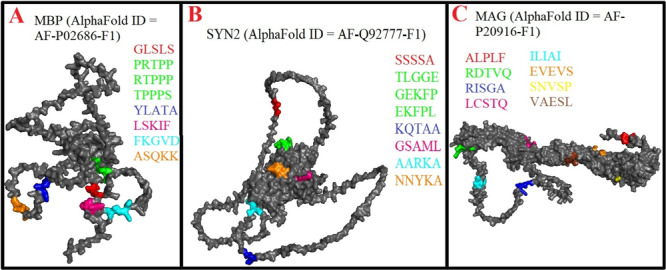
(A–C). Location of Human herpesvirus 6 (HHV‐6) mimicking pentapeptides in 3D structures of myelin basic protein (MBP), synapsin 2 (SYN2), myelin‐associated glycoprotein (MAG).

The present study identified 54 mimicry pentapeptides shared between HHV‐6 viral antigens and CNS antigens. As shown in Table 2 and Figure 4A, MBP exhibited three mimicry pentapeptides with the U24 protein and one with each of the MCP, RIR1, U38, U53, and gH. Likewise, Table 2 and Figure 4B indicate that SYN2 displayed three mimicry pentapeptides with immediate‐early protein 2 and one with each of MCP, U11, U31, U21, and U51. Meanwhile, Table 2 and Figure 4C illustrate that MAG contained two mimicry pentapeptides with MCP and U11 alongside one with each of immediate‐early protein 2, RIR1, U53, U31, gH, U100, and U12.

**Figure 5:**
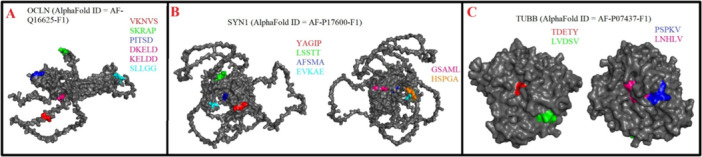
(A–C). Location of Human herpesvirus 6 (HHV‐6) mimicking pentapeptides in 3D structures of occludin (OCLN), synapsin 1 (SYN1), tubulin beta (TUBB).

**Figure 6:**
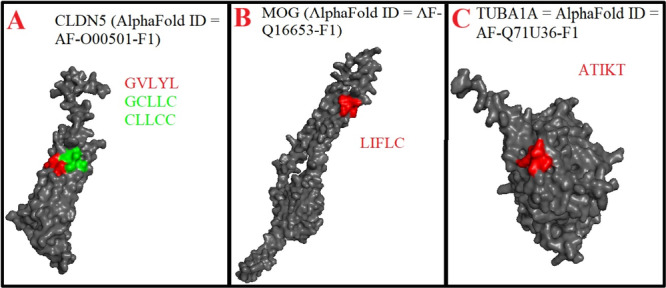
(A–C). Location of Human herpesvirus 6 (HHV‐6) mimicking pentapeptides in 3D structures of claudin 5 (CLDN5), myelin oligodendrocyte glycoprotein (MOG), tubulin alpha (TUBA1A).

Similarly, Table 2 and Figure 5A reveal that OCLN shared two mimicry pentapeptides with immediate‐early protein 2 and RIR1, as well as one with MCP and U94 protein. Table 2 and Figure 5B demonstrate that SYN1 exhibited six mimicry pentapeptides, including one with each of the 120 kDa U47, U38, gH, U21, glycoprotein 105, and U94 protein. In addition, Table 2 and Figure 5C show that TUBB contained four mimicry pentapeptides, two with DBP and one each with gB and U81. Moreover, Table 2 and Figure 6A highlight that CLDN5 displayed two mimicry pentapeptides with the U83 and one with the U38. As depicted in Table 2 and Figure 6B, MOG exhibited one mimicry pentapeptide with glycoprotein 105, while TUBA1A contained one mimicry pentapeptide with the U47.

### 3.2 The Immunogenic Potentials of Shared Pentapeptides to Trigger T and B Cells

#### 3.2.1 Immunogenic Potential of EBV‐Derived Shared Pentapeptides

The immunoreactive epitopes of EBV, which share pentapeptide sequences with CNS proteins, are summarized in Table 3. The findings demonstrate that the homologous pentapeptides present in EBV nuclear antigens (EBNA1, EBNA2, and EBNA6), latent membrane proteins (LMP1 and LMP2), early antigen D (EA‐D), and BLLF1 exhibit structural similarity with CNS autoantigens. These viral antigens can serve as immunoreactive epitopes targeted by both human T and B cells. Notably, all examined viral antigens, except for EBNA2, also share similar immunogenic pentapeptide sequences with SYN1. However, most of viral antigens demonstrated similar immunogenic pentapeptides with other CNS proteins such as SYN2 and several key myelin‐associated proteins, including MBP, MOG, and MAG, as well as the less common MOBP and CLDN5.

**Table 3:** Immunoreactive Epstein–Barr virus (EBV), Human herpesvirus 6 (HHV‐6)‐derived epitopes containing shared pentapeptides with central nervous system (CNS) proteins. Shared pentapeptides are shown with bold.

Virus	Viral Antigens	Epitope ID	T cell epitopes*	Epitope ID	B cell epitopes#	Shared CNS protein epitopes
**EBV**	EBNA1	55298	rp**QKRPS**ci	695822	**PRHRD**gvrrpqkrps	MBP*
		69558	v**LKDAI**kdl	696234	rp**QKRPS**cigckgth	MOG*, MBP#
		3579	a**PGPGP**qpgplresivcyfm	694927	ifaeg**LKDAI**kdlvl	SYN1*, MOG#
		3579	ap**GPGPQ**pgplresivcyfm	696293	rtt**DEGTW**vagvfvy	SYN1*, MAG#
		49056	**PQPGP**lresivcyfmvflqt	696293	rttd**EGTWV**agvfvy	SYN1*, MAG#
		2257600	sqssssgspp**RRPPP**grrpf	106067	r**GGSGG**rgr**GGSGG**	SYN2*, SYN1#
				227041	a**PGPGP**qpgplresi	SYN1#
				227041	ap**GPGPQ**pgplresi	SYN1#
				694695	gpg**PQPGP**lresivc	SYN1#
				696509	sssgspp**RRPPP**grr	SYN2#
	EBNA2	46923	paqp**PPGVI**ndqqlhhlpsg	2256565	padldesw**DYIFE**ttespssdedyvegpskrprpsiq	OCLN*, SYN2#
	LMP1	39681	lssslgla**LLLLLL**allfwl	693789	agnd**GGPPQ**lteeve	MOG*, SYN1#
		98685	agnd**GGPPQ**	2257473	lteevenkggd**QGPPL**mtdg	SYN1*, SYN1#
	LMP2	20892	glgt**LGAAL**	2257521	pfnsllfall**AAAGG**lqgiy	CLDN5*, SYN1#
				696976	wlsspgglgt**LGAAL**	CLDN5#
	EA‐D	—	—	750156	**PPPPP**rtp	SYN1#, SYN2#
	BLLF1	—	—	695398	lt**LLLLL**vmadcafr	MOG#
				695534	ncnstnit**AVVRA**qg	MOBP#
				695435	lyvfysgng**PKASG**g	SYN1#
	EBNA6	51245	qkr**AAPPT**vspsdtg	695726	pkpvpvkp**TPPPS**rr	SYN2*, MBP#
		48737	ppa**AGPPA**agprila	227517	rlqdirr**GPLVA**egg	SYN1*, MOG#
				696588	tgehiqd**PGPGP**stg	SYN1#
				693727	**AAPPT**vspsdtgppa	SYN1#
				693712	a**AGPPA**agphiltpp	SYN1#
				695433	lypqplv**SSSAP**sws	SYN2#
**HHV‐6**	Protein U94	17162	fniik**SLLGG**	—	—	OCLN*

Abbreviations: BLLF1, envelope glycoprotein GP350; CLDN5, claudin‐5; EA‐D, early antigen diffused; EA‐R, early antigen protein R; EBNA, Epstein–Barr nuclear antigen; gB, envelope glycoprotein B; gE, envelope glycoprotein E; gH, envelope glycoprotein H; gI, glycoprotein I; LMP, latent membrane protein; MBP, myelin basic protein; MCP, major capsid protein; MOBP, myelin‐associated oligodendrocyte basic protein; MOG, myelin‐oligodendrocyte glycoprotein; OCLN, occludin; SYN, synapsin; TUBA1A, tubulin alpha‐1A chain; TUBB, tubulin beta chain.

* shared pentapeptide is immunogenic to T cells.

^#^ shared pentapeptide is immunogenic to B cells, Bold: indicate shared pentapeptide between immune cell and human CNS protein.

#### 3.2.2 Immunogenic Potential of HHV‐6‐Derived Shared Pentapeptides

Table 3 presents the immunoreactive epitopes of HHV‐6 that exhibit shared pentapeptide sequences with CNS proteins. Protein U94 exhibiting structural similarities with CNS proteins SYN1 and OCLN, serves as an immunoreactive epitope targeted by human T cells. All other HHV‐6 derived shared pentapeptides did not function as immunoreactive epitopes for T or B cells.

## 4 Discussion

### 4.1 Shared Pentapeptides Between EBV and HHV‐6 Antigens and Host Cell CNS Epitopes

The first key finding of the present study identified molecular mimicry between 12 EBV antigens (EBNA1, EBNA2, EBNA6, LMP1, LMP2A/LMP2B, EA‐D, EA‐R, gB, gH, MCP, gE and BLLF1) and 20 HHV‐6 antigens (immediate‐early protein 2, MCP, RIR1, U24, U47, U11, DBP, U83, U38, U53, U31, gH, U21, U100, gB, U12, glycoprotein 105, U81, U51, and U94) with 10 critical CNS proteins. These include myelin proteins (MBP, MOG, MAG, and MOBP), BBB tight junction proteins (OCLN and CLDN5), cytoskeletal proteins (TUBB, and TUBA1A), and synaptic proteins (SYN1 and SYN2). This mimicry suggests that immune responses to viral antigens may cross‐react with structurally similar CNS self‐epitopes, triggering immune‐mediated CNS damage and contributing to autoimmunity. Unlike previous studies that focused on individual proteins, such as EBNA1 mimicry with GlialCAM [26], our study uniquely identifies mimicry with an expanded range of CNS proteins, including SYN1 and SYN2, and BBB‐specific proteins OCLN and CLDN5. This enhances the comprehension of the range and variety of viral‐induced autoimmune targets, especially in conditions characterized by impaired BBB and synaptic damage.

A pentapeptide represents one of 3.2 million potential combinations of amino acids (20⁵), making it statistically uncommon for two unrelated proteins to exhibit identical motifs. The identification of patterns between viral proteins and CNS autoantigens, particularly when supported by structural localization and predicted immunogenicity, suggests that these similarities are unlikely to be coincidental.

The immune mechanisms underlying this mimicry involve cross‐reactive T cells and cross‐reacting autoantibodies that damage host cell CNS epitopes and compromise the integrity of the BBB, facilitating the infiltration of peripheral immune cells into the CNS [42]. Such disruptions are hallmark features of CNS autoimmune diseases, particularly MS [42, 43] and are also prevalent in depression and CFS/ME (see Introduction).

Molecular mimicry likely functions via antigen presentation by MHC‐II molecules, wherein viral shared motifs with host cell epitopes are displayed to autoreactive T cells, resulting in pathogenic immune activation [1, 6]. The subsequent upregulation of proinflammatory cytokines, including interferon‐γ and tumor necrosis factor‐α, exacerbate neuroinflammation and contributes to demyelination [44, 45]. EBV reactivation has been shown to expand autoreactive B and T cell populations, amplifying neuroinflammation and contributing to neuronal damage [46]. Similarly, HHV‐6 latency within glial cells may induce proinflammatory cytokine release upon reactivation, further perpetuating neural injury [47]. These findings suggest that repeated viral reactivation or chronic exposure sustains inflammatory pathways and autoimmunity, offering a plausible mechanism for disease progression.

This mimicry has broader implications beyond MS, depression and CFS/ME. Research indicates that EBV and HHV‐6 may play a role in various autoimmune and neurodegenerative diseases, including neuromyelitis optica, autoimmune encephalitis, and possibly Alzheimer's disease, wherein viral reactivation has been linked to amyloid‐β pathology [48, 49, 50]. The associations highlight the systemic influence of herpesviruses in neuroinflammatory and neurodegenerative disorders. Comparative analyzes of other viral infections demonstrate unique mechanisms of molecular mimicry in CNS autoimmunity. For example, SARS‐CoV‐2 nucleocapsid protein shares homology with MS‐associated proteins, such as PLP and neurofilament light polypeptide, implicating molecular mimicry in COVID‐19‐related neuroinflammation [51]. In contrast, EBV and HHV‐6 exhibit mimicry with distinct CNS targets, including synaptic and cytoskeletal proteins, suggesting alternative pathways in autoimmune pathogenesis. The observed differences highlight the need for pathogen‐specific strategies in therapeutic development, focusing on the unique mimicry profiles of individual viruses.

Viral mimicry targeting synaptic proteins (SYN1 and SYN2) and tubulins may contribute to synaptic dysfunction, altered plasticity, and disrupted network connectivity, processes repeatedly implicated in depressive symptomatology [52, 53, 54]. These observations support a mechanistic link between viral exposure and mood disorders, including depression [55], although experimental validation remains necessary.

The identification of viral antigens that mimic key host cell CNS proteins has substantial therapeutic implications. Neutralizing monoclonal antibodies targeting viral antigens, such as EBNA1, could mitigate cross‐reactive immune responses while preserving host tissue integrity [56]. Antiviral therapies that suppress EBV and HHV‐6 reactivation could provide dual benefits by reducing viral load and dampening inflammation. Deleting EBV‐infected cells while preserving healthy tissues are under investigation and may offer a promising strategy to reduce autoimmune activation without extensive immunosuppression [57]. Additionally, the design of vaccines that exclude mimicry‐prone epitopes could prevent vaccine‐induced autoimmunity while enhancing protective immunity [57]. Therapeutic interventions that specifically target some of the identified mimicking pentapeptide epitopes are rare. These findings should be considered exploratory, offering a mechanistic framework that may inform future research focused on therapeutic development or biomarker identification.

The concept of molecular mimicry is not confined to viruses but extends to bacterial pathogens, reinforcing their role as a universal mechanism in autoimmune diseases. For instance, *Campylobacter jejuni* lipooligosaccharides mimic human gangliosides, triggering Guillain‐Barré syndrome through an autoimmune attack on peripheral nerves [58]. Likewise, antigens from *Acanthamoeba castellanii* mimic myelin proteins, such as MBP and PLP, inducing experimental autoimmune encephalomyelitis (EAE), a murine model of MS [59]. Future research must validate these findings in animal models of neuropsychiatric disease, employ peptide‐based methods to confirm cross‐reactivity, and evaluate their clinical significance in larger cohorts of patients with CNS disorders and autoimmune disorders. These efforts may establish molecular mimicry as a key mechanism connecting viral infections to autoimmune pathogenesis and inform the development of precision‐targeted interventions.

### 4.2 T and B Cells Immunogenicity of the Shared Pentapeptide Motifs

The second major finding of this study indicates that shared pentapeptides between EBV/HHV‐6 antigens and human CNS proteins may elicit potent T‐ and B‐cell‐mediated immune responses. The SYN1 and SYN2 self‐epitopes, which demonstrate shared pentapeptides homology with various EBV antigens, were identified as significant stimulators of immune activation among the shared proteins. This is consistent with recent findings that emphasize the significance of synaptic proteins in autoimmune diseases characterized by neuroinflammation and synaptic dysfunction as key features of the disease pathology [60].

Previous research indicates that MS and the affective and CFS/ME symptoms due to MS are linked to autoimmune responses involving IgA, IgG, and IgM antibodies that target critical myelin proteins, including MBP, MOG, and MAG [10]. This study extends existing evidence by proposing that autoimmune responses may be partially influenced by increased immune reactivity to viral antigens, such as EBNA and deoxyuridine‐triphosphate nucleotidohydrolases from EBV and HHV‐6 [10]. In Long COVID, antibodies directed against HHV‐6 epitopes correlate with elevated IgA and IgG titters against zonulin, a crucial protein for intestinal barrier integrity [12]. This cross‐reactivity highlights the systemic effects of molecular mimicry and its possible involvement in sustaining neuroinflammatory and autoimmune mechanisms.

The immunogenicity of shared epitopes is further supported by Begum et al. (2022), who identified 13 T‐cell epitopes shared between viral and human proteins, capable of binding promiscuously to human HLA class II alleles [61]. As mentioned earlier in this study, these epitopes likely trigger autoreactive T cells through mechanisms of molecular mimicry, which is well‐documented in autoimmune diseases such as MS [42]. Human coronaviruses and other pathogens have been implicated in activating myelin‐reactive T cells by presenting antigens that resemble CNS proteins [8, 62]. Similarly, in HAM/TSP, molecular mimicry has been shown to involve cross‐reactive antibodies targeting HTLV‐1‐tax and hnRNP A1, a neuronal protein, contributing to CNS damage [63]. EBV encodes multiple proteins that may disrupt immune tolerance by mimicking host immune‐regulatory genes. For instance, BCRF1, an IL‐10 homolog, modulates cytokine production, while BZLF‐1, a mimic of AP‐1 and NF‐κB, promotes proinflammatory pathways [64]. Moreover, exosomes released from EBV‐infected B cells contain viral components capable of triggering systemic and CNS‐specific immune responses, contributing to the development of MS [65]. These mechanisms are complemented by herpesviruses' ability to reduce epitope diversity and enhance similarity to host peptides, thereby evading immune detection and facilitating chronic infection [66]. Such strategies allow these viruses to persist in the host while simultaneously driving autoimmune pathology through prolonged immune activation.

CD8⁺ T cells recognize specific EBV antigens, such as EBNA1, presented via MHC class I molecules. For example, EBNA1 peptides restricted by HLA‐B8, HLA‐B3501, and HLA‐Cw0303 elicit strong T‐cell responses, although the glycine‐alanine repeat (GAr) domain in EBNA1 inhibits proteasomal degradation, limiting peptide availability for presentation [67, 68]. Despite this, epitopes flanking the GAr domain remain accessible and are sufficient to activate CD8⁺ T cells [67]. Likewise, CD4⁺ T cells recognize EBNA1 peptides presented by MHC class II molecules, although the protein's low expression often limits efficient presentation [69]. Nevertheless, specific epitopes, such as EYHQEGGPD, are effectively presented and induce robust CD4⁺ T‐cell responses, highlighting their potential as therapeutic targets in immunotherapy [69].

MBP‐specific T cells can be activated by viral antigens, such as EBV LMP1, through shared epitopes, promoting epitope spreading and enhancing autoreactive B‐cell responses [70]. This mechanism is critical in MS, where chronic viral reactivation redirects immune responses toward myelin proteins, amplifying neuroinflammation [71]. Similarly, MOG peptides bind efficiently to both MHC class I and II molecules, eliciting strong T‐cell responses in inflammatory demyelinating diseases [72, 73]. Antigens from EBV and HHV‐9, including dUTPase, activate the TLR2 and NFκB signaling pathways, thereby amplifying inflammatory responses that contribute to autoimmune activation [74]. Shared epitopes between EBNA1 and CNS proteins, such as alpha‐crystallin B (CRYAB), facilitate cross‐reactive T‐cell responses that are implicated in MS and other neuroinflammatory disorders [75]. Additionally, herpesviruses exploit latency phases to express immune‐evasive proteins that mimic host peptides, promote chronic infection and sustained immune activation [66, 76]. This dynamic interplay between immune evasion and mimicry establishes herpesviruses as significant contributors to autoimmune pathogenesis.

## 5 Limitations

The in‐silico approach, although effective for predicting molecular interactions and sequence homology, fails to capture the full complexity of host‐pathogen interactions in vivo. The immune responses elicited by mimicry require validation in animal models or human clinical samples. A further limitation is the insufficient data regarding the temporal relationship between viral reactivation and the onset or progression of autoimmune neuroinflammatory diseases, necessitating longitudinal studies. The integration of advanced technologies, including single‐cell RNA sequencing and high‐resolution imaging, may enhance the understanding of the cellular and molecular mechanisms underlying mimicry‐induced pathologies [77]. These approaches will enhance the evidence base and promote the application of mimicry‐based findings in clinical practice. The current analysis of mimicry was performed at the level of linear amino acid sequences, excluding the potential influence of post‐translational modifications on epitope recognition. We concentrated solely on HHV‐6A; the exclusion of HHV‐6B, which demonstrates different immunological characteristics, may limit the breadth of the conclusions drawn. Further research should focus on these elements to attain a more comprehensive assessment of herpesvirus‐related molecular mimicry.

## 6 Conclusion

This study identifies a significant overlap of pentapeptides between EBV and HHV‐6 antigens and key CNS proteins, underscoring a potential mechanism by which viral infections contribute to CNS autoimmunity through molecular mimicry. The structural similarity between these viral and host cell epitopes may elicit robust immunogenic responses from both T and B cells, suggesting that immune cross‐reactivity plays a critical role in the pathogenesis of CNS diseases. These findings reinforce the hypothesis that persistent viral infections or reactivation events may drive autoimmunity by priming autoreactive lymphocytes against self‐antigens, ultimately leading to neuroinflammatory damage. Given the implications of these interactions in neuropsychiatric disease, further investigations are warranted to delineate the precise molecular pathways involved and to explore targeted therapeutic strategies that could mitigate virus‐driven autoimmune responses.

## Author Contributions

A.F.A. and M.G.N. designed the current study. Data collection and validation, including protein and peptide codes, were performed by A.F.A., T.S., and M.G.N. Bioinformatics analyzes were conducted by M.G.N. A.F.A. and M.M. prepared the first draft of the manuscript. All authors contributed to manuscript revision and approved the final version for submission.

## Funding

The authors have nothing to report.

## Ethics Statement

The authors have nothing to report.

## Conflicts of Interest

The authors declare no conflicts of interest.

## Supporting information


**Supporting File:** iid370445‐sup‐0001‐STROBE_checklist_cohort.docx.

## Data Availability

MGN will reply to reasonable requests for the dataset used in the current study after all authors have fully utilized the data.
